# Associations Between Cardiovascular Health and Health-Related Quality of Life, Behavioral Risk Factor Surveillance System, 2013

**DOI:** 10.5888/pcd13.160073

**Published:** 2016-07-28

**Authors:** Erika C. Odom, Jing Fang, Matthew Zack, Latetia Moore, Fleetwood Loustalot

**Affiliations:** Jing Fang, Matthew Zack, Latetia Moore, Fleetwood Loustalot, National Center for Chronic Disease Prevention and Health Promotion, Centers for Disease Control and Prevention, Atlanta, Georgia.

## Abstract

**Introduction:**

The American Heart Association established 7 cardiovascular health metrics as targets for promoting healthier lives. Cardiovascular health has been hypothesized to play a role in individuals’ perception of quality of life; however, previous studies have mostly assessed the effect of cardiovascular risk factors on quality of life.

**Methods:**

Data were from the 2013 Behavioral Risk Factor Surveillance System, a state-based telephone survey of adults 18 years or older (N = 347,073). All measures of cardiovascular health and health-related quality of life were self-reported. The 7 ideal cardiovascular health metrics were normal blood pressure, cholesterol, body mass index, not having diabetes, not smoking, being physically active, and having adequate fruit or vegetable intake. Cardiovascular health was categorized into meeting 0–2, 3–5, or 6–7 ideal cardiovascular health metrics. Logistic regression models examined the association between cardiovascular health, general health status, and 3 measures of unhealthy days per month, adjusting for age, sex, race/ethnicity, education, and annual income.

**Results:**

Meeting 3 to 5 or 6 to 7 ideal cardiovascular health metrics was associated with a 51% and 79% lower adjusted prevalence ratio (aPR) of fair/poor health, respectively (aPR = 0.49, 95% confidence interval [CI] [0.47–0.50], aPR = 0.21, 95% CI [0.19–0.23]); a 47% and 72% lower prevalence of ≥14 physically unhealthy days (aPR = 0.53, 95% CI [0.51–0.55], aPR = 0.28, 95% CI [0.26–0.20]); a 43% and 66% lower prevalence of ≥14 mentally unhealthy days (aPR = 0.57, 95% CI [0.55–0.60], aPR = 0.34, 95% CI [0.31–0.37]); and a 50% and 74% lower prevalence of ≥14 activity limitation days (aPR = 0.50, 95% CI [0.48–0.53], aPR = 0.26, 95% CI [0.23–0.29]) in the past 30 days.

**Conclusion:**

Achieving a greater number of ideal cardiovascular health metrics may be associated with less impairment in health-related quality of life.

**MEDSCAPE CME**

Medscape, LLC is pleased to provide online continuing medical education (CME) for this journal article, allowing clinicians the opportunity to earn CME credit.

This activity has been planned and implemented in accordance with the Essential Areas and Policies of the Accreditation Council for Continuing Medical Education through the joint providership of Medscape, LLC and *Preventing Chronic Disease*. Medscape, LLC is accredited by the ACCME to provide continuing medical education for physicians.

Medscape, LLC designates this Journal-based CME activity for a maximum of 1 ***AMA PRA Category 1 Credit(s)™***. Physicians should claim only the credit commensurate with the extent of their participation in the activity.

All other clinicians completing this activity will be issued a certificate of participation. To participate in this journal CME activity: (1) review the learning objectives and author disclosures; (2) study the education content; (3) take the post-test with a 75% minimum passing score and complete the evaluation at http://www.medscape.org/journal/pcd; (4) view/print certificate.

Release date: July 28, 2016; Expiration date: July 28, 2017

**Learning Objectives**

Upon completion of this activity, participants will be able to:

1. Evaluate the association of ideal CVHMs with overall HRQoL, based on findings of a state-based telephone survey
2. Evaluate the association of ideal CVHMs with unhealthy days, based on findings of a state-based telephone survey
3. Determine the clinical implications of findings from this state-based telephone survey regarding the link between cardiovascular health and quality of life

**EDITOR**

Teresa Ramsey

Editor, *Preventing Chronic Disease*

Disclosure: Teresa Ramsey has disclosed no relevant financial relationships.

**CME AUTHOR**

Laurie Barclay, MD

Freelance writer and reviewer, Medscape, LLC

Disclosure: Laurie Barclay, MD, has disclosed the following relevant financial relationships:

Owns stock, stock options, or bonds from: Pfizer

**AUTHORS**

Erika C. Odom, PhD

Division for Heart Disease and Stroke Prevention, National Center for Chronic Disease Prevention and Health Promotion, Centers for Disease Control and Prevention, Atlanta, Georgia

Disclosure: Erika C. Odom, PhD, has disclosed no relevant financial relationships.

Jing Fang, MD

Division for Heart Disease and Stroke Prevention, National Center for Chronic Disease Prevention and Health Promotion, Centers for Disease Control and Prevention, Atlanta, Georgia

Disclosure: Jing Fang, MD, has disclosed no relevant financial relationships.

Matthew Zack, MD

Division of Population Health, National Center for Chronic Disease Prevention and Health Promotion, Centers for Disease Control and Prevention, Atlanta, Georgia

Disclosure: Matthew Zack, MD, has disclosed no relevant financial relationships.

Latetia Moore, PhD

Division of Nutrition, Physical Activity, and Obesity, National Center for Chronic Disease Prevention and Health Promotion, Centers for Disease Control and Prevention, Atlanta, Georgia

Disclosure: Latetia Moore, PhD, has disclosed no relevant financial relationships.

Fleetwood Loustalot, PhD

Division for Heart Disease and Stroke Prevention, National Center for Chronic Disease Prevention and Health Promotion, Centers for Disease Control and Prevention, Atlanta, Georgia

Disclosure: Fleetwood Loustalot, PhD, has disclosed no relevant financial relationships.

## Introduction

Cardiovascular disease (CVD) is the leading cause of death in the United States, accounting for about 1 in 3 deaths annually (1). The economic burden of CVD accounts for over $120 billion per year in lost productivity costs from premature illness and death (2). However, CVD mortality rates have declined during the past 4 decades. Nationally, about half of the downward shift from 1980 to 2000 in coronary heart disease, a major subcategory of heart disease, was attributable to population declines in CVD risk factors and improved health behaviors (3). The primary risk factors for CVD are well known and in 2010, the American Heart Association (AHA) collated these risk factors into a composite measure of cardiovascular health, known as Life’s Simple 7 (4). The 7 cardiovascular health metrics (CVHM) include body mass index (BMI), smoking status, physical activity, a measure of dietary intake, glucose, blood pressure, and cholesterol. Poor, intermediate, and ideal ranges were developed for each of the 7 CVHM. Findings from large, prospective studies consistently indicate that individuals with a higher number of ideal CVHMs have lower risk of ischemic heart disease, cardiovascular mortality, and all-cause mortality than individuals with 0 to 1 ideal CVHMs (5,6). Yet, fewer than 3.5% (range, 0.1%–3.3%) of US adults have ideal levels of all 7 CVHM (5–7).

Although CVD events like heart failure, heart attack, and stroke are the typical measures of illness examined in studies linking the CVHM and health outcomes, health-related quality of life (HRQOL) is also an important measure of cardiovascular illness (8–10). HRQOL indicates patients’ perceptions of their general, physical, and mental health status and describes health burden in a population. Many studies suggest that people with at least one CVD risk factor, such as diabetes, hypertension, high cholesterol, or obesity, report less than “good” HRQOL (8,9); however, less is known about the association between cardiovascular health and HRQOL (10). The primary objective of this investigation was to determine the association between 7 ideal CVHMs and HRQOL (ie, self-reported general health status and 3 measures of unhealthy days) in the 2013 Behavioral Risk Factor Surveillance System (BRFSS).

## Methods

We used data from the 2013 BRFSS, an ongoing, state-based telephone survey of the noninstitutionalized US population aged ≥18 years (N = 491,773; weighted N = 246,024,416). Participants are selected using a stratified, multistage probability sampling design, incorporating random-digit–dialing methods for data collection via both landline and cellular telephones. BRFSS uses iterative proportional fitting to improve representation of the respondents to the entire US adult population based on sex, age, race/ethnicity, county, region, telephone service (landline, cellular phone, or both), tenure (renting or owning a home), marital status, and education (11). In 2013, the median state response rate was 46.4%, with a range of 29.0% to 60.3% across different states (12). After excluding individuals with missing data on any outcome, predictor, or control measure (except income), a total analytic sample of 347,073 (71%; weighted n = 156,525,839) remained. Compared with excluded individuals, a higher proportion of people in the analytic sample had a high income (44% vs 25%, >$50k), were more educated (30% vs 18%, college educated), were older (22.5% vs 11.8%, 65 or older), and less racially/ethnically diverse (69.1% vs 56.8% white).

HRQOL was measured using 4 self-reported indicators: 1) general health status (“Would you say that in general your health is excellent, very good, good, fair, or poor?”); 2) physically unhealthy days per month (“Now thinking about your physical health, which includes physical illness and injury, for how many days during the past 30 days was your physical health not good?”); 3) mentally unhealthy days per month (“Now thinking about your mental health, which includes stress, depression, and problems with emotions, for how many days during the past 30 days was your mental health not good?”); and 4) days per month of activity limitation (“During the past 30 days, for about how many days did poor physical or mental health keep you from doing your usual activities, such as self-care, work, or recreation?”). The HRQOL indicators and their validity have been extensively described (13). We dichotomized responses for general health status as “fair/poor” or “excellent/very good/good.” Additionally, we dichotomized each of the remaining indicators into mutually exclusive groups depending on whether an individual reported 14 or more unhealthy days or fewer than 14 unhealthy days (α = 0.70). Previous studies define 14 or more unhealthy days as a meaningful cut point for those reporting substantially impaired HRQOL (8,14). We also calculated average unhealthy days, indicating the mean number of physically or mentally unhealthy days per month (ie, a maximum of 30 days).

Proxy indicators for CVHM included participant self-report of BMI, current smoking status, physical activity, consumption of fruits or vegetables, diabetes, high blood pressure, and high cholesterol (Appendix). Relevant BRFSS questions for the CVHMs used in this study were based on AHA standards and have been used in previous analyses (4,7). Responses for each of the 7 CVHM were dichotomized as ‘0’ for not meeting the ideal or ‘1’ for meeting the ideal status for that individual metric and were based on self-report. The seven CVHM are summed for a score, with a range of 0 to 7. For the purposes of this study, we created a 3-level categorical score of cardiovascular health (CVH composite score) to indicate meeting ideal status on 0 to 2, 3 to 5, and 6 to 7 CVHM. Ideal smoking status included those who had not smoked at least 100 cigarettes during their lifetime or who smoked at least 100 cigarettes during their lifetime but were not currently smoking. Ideal physical activity included meeting weekly aerobic recommendations of 150 or more minutes of moderate-intensity activity, or 75 or more minutes of vigorous intensity activity, or an equivalent combination. Fruit and vegetable intake was reported via a 6-item screener on consumption of 100% fruit juice, whole fruit, dried beans, dark green vegetables, orange vegetables, and other vegetables during the previous month. Individuals were classified as having an ideal diet if their consumption met or exceeded age- and sex-specific federal fruit or vegetable intake recommendations for those with a sedentary lifestyle (15). The indicators for hypertension, high cholesterol, and diabetes were categorized as “no” (ideal) or “yes” based on self-report.

Sociodemographic control variables included age group (≥18–24, 25–34, 35–44, 45–54, 55–64, ≥65), sex (male, female), race/ethnicity (non-Hispanic whites, non-Hispanic blacks, non-Hispanic Asian, non-Hispanic American Indian/Alaskan Natives, Hispanics, non-Hispanic persons of other races), education (<high school diploma, high school diploma, some college, ≥college graduate), and annual household income (<$25K, ≥$25K to $50K, >$50K).

We estimated the prevalence and 95% confidence intervals (CIs) of HRQOL measures for selected sociodemographic characteristics, the 7 CVHM, and the CVH composite score. Prevalence estimates were age-standardized to the 2000 US standard population, except for those associated with specific age groups. Finally, we used multiple logistic regression to examine the association between meeting ideal cardiovascular health and the likelihood of reporting poor general health and each measure of unhealthy days, adjusting for age, sex, education, race, and income. About 10% of the BRFSS survey sample had missing information on income; therefore, a “missing” category was created for the income variable (ie, <$25K, ≥$25K-$50K, >$50K, missing). There were 8 logistic regression models for general health status and each measure of unhealthy days — 7 for each CVHM as the primary predictor and 1 for the CVH composite score as the primary predictor. For all models, we estimated model-adjusted prevalence ratios (aPR) on average marginal predictions (16). Because multiple comparisons were made on the HRQOL variables, we used the Bonferroni correction, the most conservative approach for declaring significance. Differences were significant if *P* < .0063. We used SAS 9.3 and SAS-callable SUDAAN (SAS Institute, Inc.) with design variables and sampling weights to account for the complex survey design.

## Results

Overall, 16.1% of adults reported their general health status as fair or poor, a proportion that increased with age, was higher in women than men, and higher in Hispanics than other racial/ethnic groups. The prevalence of fair/poor health decreased with higher education and income. Similar patterns occurred by sex, education, and income across the remaining measures of HRQOL. The overall prevalence for 14 or more physically unhealthy days was 11.3%; 14 or more mentally unhealthy days, 10.8%; and 14 or more activity limitation days, 7.6% (Table 1). Unlike the other unhealthy days indicators, the percentage of adults with 14 or more mentally unhealthy days generally decreased with age.

**Table 1:** Age-Standardized^a^ Percentages of Health-Related Quality of Life Measures by Sociodemographic Characteristics, 2013 Behavioral Risk Factor Surveillance System

Characteristics	Total Sample	Health Status Fair/Poor	≥14 Physically Unhealthy Days	≥14 Mentally Unhealthy Days	≥14 Activity Limitation Days
	Unweighted N (%)	%^b^ (95% CI)	%^b^ (95% CI)	%^b^ (95% CI)	%^b^ (95% CI)
Overall	347,073 (100)	16.1 (15.8–16.4)	11.3 (11.1–11.5)	10.8 (10.5–11.0)	7.6 (7.4–7.8)
**Age, y**					
18–24	8,681 (6.7)	8.2 (7.3–9.2)	5.1 (4.4–5.9)	11.6 (10.6–12.7)	3.5 (2.9–4.3)
25–34	25,529 (13.3)	10.3 (9.6–11.1)	6.7 (6.1–7.2)	11.4 (10.7–12.2)	5.1 (4.6–5.7)
35–44	40,823 (16.7)	13.3 (12.6–13.9)	9.7 (9.2–0.3)	10.9 (10.3–11.5)	7.2 (6.7–7.7)
45–54	63,600 (21.1)	18.5 (17.9–19.1)	14.0 (13.5–14.6)	12.9 (12.4–13.4)	10.1 (9.6–10.5)
55–64	84,522 (19.8)	22.3 (21.7–23.0)	16.3 (15.8–16.8)	11.6 (11.2–12.1)	11.1 (10.6–11.5)
≥65	123,918 (22.5)	24.4 (23.9–24.9)	16.1 (15.7–16.5)	6.7 (6.5–7.0)	8.7 (8.4–9.0)
**Sex**					
Male	143,113 (48.5)	15.1 (14.7–15.5)	10.0 (9.6–10.3)	8.5 (8.2–8.9)	6.6 (6.3–6.9)
Female	20,3960 (51.5)	17.1 (16.7–17.5)	12.6 (12.2–12.9)	13.0 (12.6–13.4)	8.6 (8.3–8.9)
**Race/Ethnicity**					
White, non-Hispanic	279,948 (69.1)	13.4 (13.1–13.7)	10.9 (10.6–11.2)	10.6 (10.4–10.9)	7.3 (7.1–7.5)
Black, non-Hispanic	25,962 (10.8)	21.7 (20.7–22.7)	12.7 (11.9–13.5)	12.2 (11.3–13.1)	9.2 (8.6–10.0)
Asian	5,942 (4.1)	10.6 (9.1–12.4)	5.7 (4.6–6.9)	6.5 (5.3–7.9)	4.0 (3.0–5.2)
American Indian/Alaskan Native	4,751 (0.9)	23.7 (21.2–26.4)	18.5 (16.2–21.1)	16.9 (14.6–19.5)	13.2 (11.3–15.4)
Hispanic	21,716 (13.2)	27.9 (26.8–29.0)	13.9 (13.1–14.7)	12.1 (11.3–13.0)	8.4 (7.7–9.2)
Other race, non-Hispanic	8,754 (1.8)	20.6 (18.8–22.5)	16.8 (15.0–18.8)	14.7 (12.9–16.7)	11.3 (9.9–12.8)
**Education**					
<High school	23,608 (11.8)	37.5 (36.1–39.0)	21.1 (20.0–22.2)	18.0 (16.9–19.3)	15.3 (14.3–16.3)
High school diploma or GED	94,828 (26.7)	19.1 (18.6–19.7)	13.0 (12.6–13.5)	12.4 (11.9–12.9)	8.9 (8.5–9.3
Some college	95,574 (31.6)	14.5 (14.1–15.0)	11.5 (11.1–11.9)	11.5 (11.0–11.9)	7.7 (7.4–8.1)
≥College graduate	133,063 (29.9)	7.0 (6.7–7.4)	6.0 (5.7–6.2)	6.2 (5.9–6.5)	3.6 (3.5–3.8)
**Income, $** c					
<25,000	82,903 (23.6)	34.5 (32.7–34.3)	21.7 (21.1–22.4)	19.9 (19.3–20.6)	16.7 (16.1–17.3)
≥25,000 to 50,000	80,397 (21.9)	15.8 (15.2–16.4)	10.8 (10.3–11.3)	10.7 (10.1–11.2)	7.0 (6.6–7.5)
>50,000	145,601 (44.2)	7.0 9(6.7–7.4)	6.0 (5.7–6.2)	6.4 (6.1–6.8)	3.2 (3.0–3.4)

Abbreviation: CI, confidence interval.

a Age standardization applied to overall, sex, race/ethnicity, and education, using 2000 US standard projected population, with age groups (18–24, 25–44, 45–64, ≥65).

b Weighted percentages presented. Weighted n = 156,525,839; unweighted n = 347,073.

c About 10% of the BRFSS survey has missing information on income. A category was created for missing income and used in analysis; however, the results are not included in this table.

For each of the 7 CVHM, the prevalence of ideal cardiovascular health ranged from 16.4% (met fruit or vegetable intake) to 87.8% (no history of diabetes; Table 2). Although 16.5% of individuals reported meeting 0 to 2 CVHM, 69.4% met 3 to 5 CVHM and 14.1% of adults met 6 or more CVHM. Only 2.4% of individuals met ideal cardiovascular health for all 7 metrics. In general, there was an inverse relationship between CVHM and HRQOL; the prevalence of poor general health or unhealthy days was 1.5 to 3 times as high among adults who reported not meeting ideal cardiovascular health for each metric (except for meeting fruit or vegetable intake; Table 2). Adults meeting 0 to 2 CVHM reported an average of 11.3 (standard error [SE], 0.21) unhealthy days per month; adults meeting 3 to 5 CVHM reported an average of 6.0 (SE, 0.05) unhealthy days; adults meeting 6 to 7 CVHM reported an average 3.6 (SE, 0.07) unhealthy days.

**Table 2:** Age-Standardized^a^ Percentages of Cardiovascular Health by Health-Related Quality of Life, 2013 Behavioral Risk Factor Surveillance System

Cardiovascular Health Metric	Total Sample	Health Status Fair/Poor	≥14 Physically Unhealthy Days	≥14 Mentally Unhealthy Days	≥14 Activity Limitation Days
	%^c^	%^c^ (95% CI)	%^c^ (95% CI)	%^c^ (95% CI)	%^c^ (95% CI)
**Normal BMI (18.5–24.9)^a^^,^ b**					
Yes	31.5	11.5 (11.1–11.9)	9.1 (8.7–9.4)	9.4 (9.0–9.8)	6.2 (5.9–6.5)
No	68.5	18.4 (18.0–18.8)	12.3 (12.0–12.6)	11.5 (11.2–11.9)	8.3 (8.0–8.5)
**Current smoker^a^**					
No	83.8	14.2 (13.9–14.5)	9.8 (9.6–10.1)	8.8 (8.5–9.1)	6.2 (6.0–6.4)
Yes	16.2	25.5 (24.7–26.3)	18.1 (17.5–18.8)	20.0 (19.2–20.7)	14.0 (13.4–14.6)
**Met physical activity goal**					
Yes	52.1	10.7 (10.4–11.1)	7.5 (7.2–7.8)	8.6 (8.3–8.9)	4.7 (4.5–5.0)
No	47.9	21.9 (21.4–22.4)	15.3 (15.0–15.7)	13.1 (12.7–13.5)	10.7 (10.4–11.0)
**Met fruit or vegetable^a^ intake**					
Yes	16.4	13.8 (13.0–14.6)	10.3 (9.8–10.9)	10.0 (9.3–10.7)	7.1 (6.6–7.7)
No	83.6	16.6 (16.3–16.9)	11.5 (11.2–11.7)	11.0 (10.7–11.2)	7.7 (7.5–7.9)
**Diabetes^a^**					
No	87.8	13.2 (12.9–13.4)	9.8 (9.6–10.)	10.1 (9.8–10.4)	6.6 (6.4–6.8)
Yes	12.2	46.5 (44.1–49.0)	25.6 (23.8–27.5)	18.1 (16.4–19.9)	17.2 (15.6–18.9)
**Hypertension**					
Yes	37.6	26.8 (25.9–27.6)	16.8 (16.2–17.4)	16.0 (15.2–16.8)	11.7 (11.1–12.3)
**High cholesterol^a^**					
No	61.2	12.7 (12.4–13.0)	9.2 (9.0–9.5)	9.0 (8.7–9.3)	6.0 (5.8–6.3)
Yes	38.8	22.9 (22.1–23.7)	14.9 (14.4–15.5)	15.0 (14.3–15.7)	10.5 (10.0–11.1)
**CVH composite score^a^**					
0–2	16.5	38.9 (37.4–40.5)	24.4 (23.2–25.7)	21.7 (20.3–23.2)	17.1 (16.1–18.2)
3–5	69.4	14.0 (13.7–14.3)	10.0 (9.7–10.2)	10.2 (9.9–10.5)	6.6 (6.4–6.8)
6-7	14.1	4.4 (4.0–4.9)	4.4 (4.1–4.8)	5.5 (5.1–6.0)	2.7 (2.4–3.1)

Abbreviations: BMI, body mass index; CI, confidence interval; CVH, cardiovascular health.

a Age-standardization applied to each cardiovascular health metric and the cardiovascular health score (CVH composite score), using 2000 US standard projected population, by age group (18-24 y, 25-44 y, 45-64 y, ≥65 y). The composite score is a 3-level categorical variable that indicates meeting ideal status on 0–2, 3–5, and 6–7 CVHM. Diabetes, hypertension, and high cholesterol based on self-reported diagnoses.

b Compared to overweight or obese individuals or people with BMI of 18.5 or less.

c Weighted percentage presented. Weighted n = 156,525,839; Unweighted n = 347,073.

The prevalence of fair/poor health status was nearly 10 times as great among adults who met only 0 to 2 CVHM than among adults who met 6 to 7 CVHMs. Similarly, compared with adults with 6 to 7 CVHM, the prevalence of 14 or more physically unhealthy days was 6 times greater among adults who met only 0 to 2 CVHM; 4 times greater among adults reporting 14 or more mentally unhealthy days and 0 to 2 CVHM; and, 8 times greater among adults reporting 14 or more activity limitation days and 0 to 2 CVHM (Table 2).

In the logistic regression models, after controlling for sociodemographic variables, meeting ideal cardiovascular health was inversely associated with HRQOL (Table 3). The association between cardiovascular health and fair/poor health ranged from a 9% lower prevalence among individuals who met fruit or vegetable intake (aPR = 0.91 vs those who did not meet fruit or vegetable intake, 95% CI [0.87–0.95]) to a 56% lower prevalence of fair/poor health among people who did not have a history of diabetes (aPR = 0.44 vs those who reported being told they have diabetes, 95% CI [0.43–0.45]). The association between cardiovascular health and 14 or more physically unhealthy days ranged from a 19% lower prevalence among individuals who reported a normal BMI (aPR = 0.81 vs those with overweight/obese BMI or BMI <18.5, 95% CI [0.78–-0.85]) to a 46% lower prevalence among individuals who did not have a history of diabetes (aPR = .54 vs those who reported being told they have diabetes, 95% CI [0.52–0.56]). The association between cardiovascular health and 14 or more mentally unhealthy days ranged from a 8% lower prevalence among individuals who met fruit or vegetable intake (aPR = 0.92 vs those who did not meet fruit or vegetable intake, 95% CI [0.86–0.97]) to a 44% lower prevalence among individuals who did not currently smoke (aPR = 0.56 vs those who reported smoking, 95% CI [0.53–0.58]). Finally, the association between cardiovascular health and 14 or more activity limitation days ranged from a 16% lower prevalence among individuals who reported a normal BMI (aPR = 0.84 vs those with overweight/obese BMI or BMI <18.5, 95% CI [0.79–0.88]) to a 49% lower prevalence among individuals reporting moderate/vigorous physical activity (aPR = 0.51 vs those who reported not meeting physical activity recommendations, 95% CI [0.48–0.53]). There was no association between meeting fruit or vegetable intake goals and 14 or more physically unhealthy days or 14 or more activity limitation days.

**Table 3:** Adjusted Prevalence Ratio of Ideal Cardiovascular Health by Health-Related Quality of Life, 2013 Behavioral Risk Factor Surveillance System

Cardiovascular Health Metric	Health Status, Fair/Poor	14 or More Physically Unhealthy Days	14 or More Mentally Unhealthy Days	14 or More Activity Limitation Days
	aPR (95% CI)^a^^,^ b			
**Normal BMI (18.5–24.9)^c^**	0.75 (0.72–0.77)^d^	0.81 (0.78–0.85)^d^	0.87 (0.83–0.91)^d^	0.84 (0.79–0.88)^d^
**Not current smoker**	0.73 (0.70–0.75)^d^	0.70 (0.68–0.73)^d^	0.56 (0.53–0.58)^d^	0.62 (0.59–0.66)^d^
**Met physical activity goal**	0.60 (0.58–0.62)^d^	0.56 (0.54–0.58)^d^	0.70 (0.67–0.73)^d^	0.51 (0.48–0.53)^d^
**Met fruit or vegetable intake**	0.91 (0.87–0.95)^d^	0.99 (0.94–1.04)	0.92 (0.86–0.97)^d^	1.00 (0.94–1.06)
**No diabetes**	0.44 (0.43–0.45)^d^	0.54 (0.52–0.56)^d^	0.69 (0.65–0.73)^d^	0.56 (0.53–0.59)^d^
**No hypertension**	0.52 (0.50–0.53)^d^	0.60 (0.58–0.62)^d^	0.65 (0.62–0.68)^d^	0.62 (0.59–0.65)^d^
**No high cholesterol**	0.65 (0.63–0.67)^d^	0.69 (0.66–0.71)^d^	0.65 (0.62–0.68)^d^	0.66 (0.63–0.69)^d^
**CVH composite score**				
0–2	1 [Reference]	1 [Reference]	1 [Reference]	1 [Reference]
3–5	0.49 (0.47–0.50)^d^	0.53 (0.51–0.55)^d^	0.57 (0.55–0.60)^d^	0.50 (0.48–0.53)^d^
6–7	0.21 (0.19–0.23)^d^	0.28 (0.26–0.30)^d^	0.34 (0.31–0.37)^d^	0.26 (0.23–0.29)^d^

Abbreviations: aPR, adjusted prevalence ratio; BMI, body mass index; CI, confidence interval; CVH, cardiovascular health.

a Adjusted for age, sex, education, income, and race/ethnicity.

b Weighted aPR presented.

c Compared with individuals considered overweight/obese (BMI ≥25) or underweight (BMI <18.5). BMI is calculated as weight in kilograms (kg) divided by height in meters squared.

d *P* < .0063 for differences reported.

Compared with adults meeting 2 or fewer CVHM, meeting 3 to 5 or 6 to 7 CVHM was associated with 51% and 79% lower prevalence of fair/poor health respectively (aPR = 0.49, 95% CI [0.47–0.50], aPR = 0.21, 95% CI [0.19–0.23]) ; 47% and 72% lower prevalence of 14 or more physically unhealthy days respectively (aPR = 0.53, 95% CI [0.51–0.55], aPR = 0.28, 95% CI [0.26–0.30]); 43% and 66% lower prevalence of 14 or more mentally unhealthy days respectively (aPR = 0.57, 95% CI [0.55–0.60], aPR = 0.34, 95% CI [0.31–0.37]); 50% and 74% lower prevalence of 14 or more activity limitation days respectively (aPR = 0.50, 95% CI [0.48–0.53], aPR = 0.26, 95% CI [0.23–0.29]) . Additionally, the adjusted prevalence ratio for meeting 6 to 7 CVHM was nearly half the adjusted prevalence ratio for meeting 3 to 5 CVHM across each of the poor HRQOL measures.

## Discussion

Our study examined the association between cardiovascular health and HRQOL measures in a population-based surveillance system. Adults meeting 0 to 2 CVHM reported an average of 11.3 unhealthy days per month; adults meeting 3 to 5 CVHM reported an average of 6.0 unhealthy days; adults meeting 6 to 7 CVHM reported an average of 3.6 unhealthy days. Compared with people meeting 0 to 2 CVHM, people meeting 6 or more CVHM were significantly less likely to report poor general health, 14 or more physically or mentally unhealthy days, and 14 or more days of activity limitation, suggesting that meeting ideal cardiovascular health recommendations may have a cumulative impact on various self-reported measures of health. Although the directionality and contributions of CVHM on HRQOL cannot be assessed, the association with cardiovascular health is evident and stable even in adjusted models.

Previous research has consistently demonstrated the benefits of improvements in cardiovascular health on traditional outcome measures, like heart disease and stroke mortality (5,6). Our results extend these findings by demonstrating the possible beneficial effect of cardiovascular health on HRQOL, another aspect of disease burden. Studies that have examined HRQOL as an outcome measure have primarily explored the association with cardiovascular risk factors (8,9,14,17,18). Yet, a recent study using the National Health and Nutrition Examination Survey (NHANES) also showed an association between cardiovascular health and quality of life ─ compared with those in poor CVH, individuals in intermediate CVH were 44% less likely to report being in fair or poor health, and individuals in ideal CVH were 71% less likely to report being in fair or poor health (19). Likewise, findings from the Look AHEAD trial, an intensive lifestyle intervention for people with type 2 diabetes, show that lifestyle modifications that support cardiovascular health (ie, including dietary changes) are associated with fewer hospitalizations, fewer medications, lower health care costs, and better quality of life, suggesting that cardiovascular health may be associated with HRQOL in ways beyond decreasing cardiovascular disease and disability (20,21).

The current study has a few limitations. The analytic sample included a higher proportion of individuals who were older, white, had more education, and a higher income than those excluded from the sample; thus, the generalizability of the findings may be limited.

Second, no clinical measures of BMI, smoking, diabetes, hypertension, or cholesterol were collected; because of this, it is possible that some participants were miscategorized as having ideal cardiovascular health and that we overestimate true prevalence. On the other hand, some participants with diabetes, hypertension, or high cholesterol may not have been diagnosed, underestimating true prevalence. Lower prevalence estimates of CVD risk factors have been consistently reported in validation studies of BRFSS when comparing them to studies with direct physical measures (22,23). Third, in the current study we estimated whether participants consumed recommended amounts of fruits or vegetables; only 3% of the participants reported meeting recommendations for both fruits and vegetables. We defined ideal diet using this more flexible classification (ie, meeting recommendations for fruit intake *or* meeting recommendations for vegetable intake) to support more stable statistical modeling; however, this methodology overestimates diet quality. Additionally, in BRFSS, diet quality is not a comprehensive measure of dietary recall. Although AHA’s healthy diet score is made on the basis of multiple components of a healthy diet (ie, intake of fruits and vegetables, whole grains, sodium, sugar-sweetened beverages, and fish), fruit and vegetable intake has been used in previous studies as a proxy for a diet supporting cardiovascular health (7,24). Finally, our study was cross-sectional and provides only a snapshot of participants’ report of cardiovascular health and HRQOL, which does not allow conclusions to be drawn about causality. Thus, although we found that having a greater number of CVHM was associated with less impairment in HRQOL, people with impaired HRQOL may be more sedentary and less likely to participate in behaviors that support cardiovascular health — decreasing one’s ability to attain ideal recommendations for the CVHM.

In conclusion, the current study findings support an association between cardiovascular health and less impairment in general health and unhealthy days. Although BRFSS has methodological limitations with respect to the traditional AHA definition of cardiovascular health, these findings are consistent with other research. By the year 2030, lost productivity costs due to cardiovascular illness and death are projected to rise to more than $275 billion (2). Promoting cardiovascular health could help improve the quality of life for all Americans by reducing the number of physically and mentally unhealthy days that individuals experience and reducing societal costs due to lost productivity. Primary care providers should continue to encourage lifestyle modifications that are heart-healthy, including meeting diet and physical activity recommendations, and use HRQOL measures as a screening tool to regularly monitor improvements or declines in self-reported health.

## Appendix Proxy Indicators for Cardiovascular Health Metrics

**Table:**

Metric	BRFSS Question	Definition for Ideal Cardiovascular Health
**Body mass index**	About how much do you weigh without shoes? About how tall are you without shoes?	BMI = 18.5–24.9. BMI is calculated as weight measured in kilograms (kg) divided by height measured in meters squared.
**Smoking status**	Have you smoked at least 100 cigarettes in your entire life? Do you now smoke cigarettes every day, some days, or not at all? During the past 12 months, have you stopped smoking for 1 day or longer because you were trying to quit smoking? How long has it been since you last smoked cigarettes regularly?	Had not smoked at least 100 cigarettes in your lifetime; or reported smoking 100 cigarettes in your lifetime but not currently smoking.
**Physical activity**	Now, thinking about the moderate activities you do in a usual week, do you do moderate activities for at least 10 min at a time, such as brisk walking, bicycling, vacuuming, gardening, or anything else that causes some increase in breathing or heart rate? How many days/week do you do these moderate activities for at least10 min at a time? On days when you do moderate activities for at least 10 min at a time, how much total time per day do you spend doing these activities? Now, thinking about the vigorous activities you do in a usual week, do you do vigorous activities for at least 10 min at a time, such as running, aerobics, heavy yard work, or anything else that causes large increases in breathing or heart rate? How many days/week do you do these vigorous activities for at least 10 min at a time? On days when you do vigorous activities for at least 10 min at a time, how much total time per day do you spend doing these activities?	Did enough moderate or vigorous physical activity to meet the recommendation of ≥150 min a week of moderate-intensity activity, ≥75 min of vigorous-intensity activity, or an equivalent combination of aerobic physical activity.
**Healthy diet**	During the past month, how many times per day, week or month did you drink 100% PURE fruit juices? During the past month, not counting juice, how many times per day, week, or month did you eat fruit? During the past month, how many times per day, week, or month did you eat cooked or canned beans, such as refried, baked, black, garbanzo beans, beans in soup, soybeans, edamame, tofu or lentils? During the past month, how many times per day, week, or month did you eat dark green vegetables for example broccoli or dark leafy greens including romaine, chard, collard greens or spinach? During the past month, how many times per day, week, or month did you eat orange-colored vegetables such as sweet potatoes, pumpkin, winter squash, or carrots? Not counting what you just told me about, during the past month, about how many times per day, week, or month did you eat OTHER vegetables?	Met sex- and age-specific cup equivalent recommendations for fruit or vegetable intake.
**Diabetes**	Have you ever been told by a doctor that you have diabetes?	Answered “no”
**Hypertension**	Have you ever told by a doctor, nurse, or other health professional that you have high blood pressure?	Answered “no”
**Cholesterol**	Those who have cholesterol screened — Have you ever been told by a doctor, nurse, or other health professional that your blood cholesterol is high?	Answered “no”

Abbreviation: BRFSS, Behavioral Risk Factor Surveillance System.

## Post-Test Information

To obtain credit, you should first read the journal article. After reading the article, you should be able to answer the following, related, multiple-choice questions. To complete the questions (with a minimum 75% passing score) and earn continuing medical education (CME) credit, please go to http://www.medscape.org/journal/pcd. Credit cannot be obtained for tests completed on paper, although you may use the worksheet below to keep a record of your answers. You must be a registered user on Medscape.org. If you are not registered on Medscape.org, please click on the "Register" link on the right hand side of the website to register. Only one answer is correct for each question. Once you successfully answer all post-test questions you will be able to view and/or print your certificate. For questions regarding the content of this activity, contact the accredited provider, CME@medscape.net. For technical assistance, contact CME@webmd.net. American Medical Association’s Physician’s Recognition Award (AMA PRA) credits are accepted in the US as evidence of participation in CME activities. For further information on this award, please refer to http://www.ama-assn.org/ama/pub/about-ama/awards/ama-physicians-recognition-award.page. The AMA has determined that physicians not licensed in the US who participate in this CME activity are eligible for ***AMA PRA Category 1 Credits™***. Through agreements that the AMA has made with agencies in some countries, AMA PRA credit may be acceptable as evidence of participation in CME activities. If you are not licensed in the US, please complete the questions online, print the AMA PRA CME credit certificate and present it to your national medical association for review.

## Post-Test Questions

### Study Title: Associations Between Cardiovascular Health and Health-Related Quality of Life, Behavioral Risk Factor Surveillance System, 2013

#### CME Questions

1. Your patient is a 46-year-old man with cardiovascular risk factors seeking advice regarding improving his overall health. According to the state-based telephone survey by Odom and colleagues, which of the following statements is ***correct*** regarding the association of ideal cardiovascular health metrics (CVHMs) with overall health-related quality of life (HRQoL)?
  - Seven ideal CVHMs were normal blood pressure, cholesterol, body mass index, absence of diabetes, not smoking, physically active lifestyle, and adequate fruit or vegetable intake
  - Meeting 3 to 5 ideal CVHMs was associated with a 25% lower adjusted prevalence (aPR) of fair to poor health
  - Meeting 6 to 7 ideal CVHMs was associated with a 51% lower aPR of fair to poor health
  - Body mass index, smoking, diabetes, hypertension, and cholesterol were verified clinically
2. According to the state-based telephone survey by Odom and colleagues, which of the following statements about the association of ideal CVHMs with unhealthy days is ***correct***?
  - Meeting 3 to 5 ideal CVHMs was associated with a 73% lower prevalence of at least 14 physically unhealthy days in the past 30 days
  - Meeting 3 to 5 or 6 to 7 ideal CVHMs was not associated with the number of mentally unhealthy days
  - Meeting 3 to 5 or 6 to 7 ideal CVHMs was associated with a 50% and 74% lower prevalence of at least 14 activity limitation days in the past 30 days, respectively
  - The findings can be applied to low-income, less-educated African Americans
3. According to the state-based telephone survey by Odom and colleagues, which of the following statements about clinical implications of findings from this state-based telephone survey is ***correct***?
  - The findings contrast dramatically with those of earlier studies
  - Lost productivity costs due to cardiovascular morbidity and mortality are likely to stabilize for the next 15 years
  - Promoting cardiovascular health is unlikely to improve overall HRQoL in the United States
  - Clinicians should encourage heart-healthy lifestyle changes (eg, diet and physical activity recommendations) and use HRQoL measures to screen and monitor changes in health

**Table:** Evaluation

**1. The activity supported the learning objectives.**				
**Strongly Disagree**				**Strongly Agree**
1	2	3	4	5
**2. The material was organized clearly for learning to occur.**				
**Strongly Disagree**				**Strongly Agree**
1	2	3	4	5
**3. The content learned from this activity will impact my practice.**				
**Strongly Disagree**	** **	** **	** **	**Strongly Agree**
1	2	3	4	5
**4. The activity was presented objectively and free of commercial bias.**				
**Strongly Disagree**				**Strongly Agree**
1	2	3	4	5

## References

[1] Go AS , Mozaffarian D , Roger VL , Benjamin EJ , Berry JD , Borden WB , ; American Heart Association Statistics Committee and Stroke Statistics Subcommittee. Heart disease and stroke statistics—2013 update: a report from the American Heart Association [published correction appears in Circulation 2013;127:e6–e245]. Circulation 2013;127(1):e6–245. 10.1161/CIR.0b013e31828124ad 23239837 PMC5408511

[2] Mozaffarian D , Benjamin EJ , Go AS , Arnett DK , Blaha MJ , Cushman M , ; American Heart Association Statistics Committee and Stroke Statistics Subcommittee. Heart disease and stroke statistics—2015 update: a report from the American Heart Association. Circulation 2015;131(4):e29–322. 10.1161/CIR.0000000000000152 25520374

[3] Ford ES , Ajani UA , Croft JB , Critchley JA , Labarthe DR , Kottke TE , Explaining the decrease in US deaths from coronary disease, 1980–2000. N Engl J Med 2007;356(23):2388–98. 10.1056/NEJMsa053935 17554120

[4] Lloyd-Jones DM , Hong Y , Labarthe D , Mozaffarian D , Appel LJ , Van Horn L , ; American Heart Association Strategic Planning Task Force and Statistics Committee. Defining and setting national goals for cardiovascular health promotion and disease reduction: the American Heart Association’s strategic Impact Goal through 2020 and beyond. Circulation 2010;121(4):586–613. 10.1161/CIRCULATIONAHA.109.192703 20089546

[5] Folsom AR , Yatsuya H , Nettleton JA , Lutsey PL , Cushman M , Rosamond WD ; Atherosclerosis Risk in Communities Study Investigators. Community prevalence of ideal cardiovascular health, by the American Heart Association definition, and relationship with cardiovascular disease incidence. J Am Coll Cardiol 2011;57:1690–6. 10.1016/j.jacc.2010.11.041 21492767 PMC3093047

[6] Yang Q , Cogswell ME , Flanders WD , Hong Y , Zhang Z , Loustalot F , Trends in cardiovascular health metrics and associations with all-cause and CVD mortality among US adults. JAMA 2012;307(12):1273–83. 10.1001/jama.2012.339 22427615 PMC9004324

[7] Fang J , Yang Q , Hong Y , Loustalot F . Status of cardiovascular health among adult Americans in the 50 States and the District of Columbia, 2009. J Am Heart Assoc 2012;1(6):e005371. 10.1161/JAHA.112.005371 23316331 PMC3540670

[8] Chen HY , Baumgardner DJ , Rice JP . Health-related quality of life among adults with multiple chronic conditions in the United States, Behavioral Risk Factor Surveillance System, 2007. Prev Chronic Dis 2011;8(1):A09. http://www.cdc.gov/pcd/issues/2011/jan/09_0234.htm. Accessed February 11, 2016. 21159221 PMC3044020

[9] Li C , Ford ES , Mokdad AH , Balluz LS , Brown DW , Giles WH . Clustering of cardiovascular disease risk factors and health-related quality of life among US adults. Value Health 2008;11(4):689–99. 10.1111/j.1524-4733.2007.00307.x 18194400

[10] Daviglus ML , Liu K , Pirzada A , Yan LL , Garside DB , Feinglass J , Favorable cardiovascular risk profile in middle age and health-related quality of life in older age. Arch Intern Med 2003;163(20):2460–8. 10.1001/archinte.163.20.2460 14609782

[11] Behavioral Risk Factor Surveillance System. 2013 comparability of data. Atlanta (GA): Centers for Disease Control and Prevention; 2013. http://www.cdc.gov/brfss/annual_data/2013/pdf/Compare_2013.pdf. Accessed August 24, 2015.

[12] Behavioral Risk Factor Surveillance System. 2013 summary data quality report. Atlanta (GA): Centers for Disease Control and Prevention; 2013. http://www.cdc.gov/brfss/annual_data/2013/pdf/2013_DQR.pdf. Accessed August 24, 2015.

[13] Measuring healthy days: population assessment of health-related quality of life. Atlanta (GA): Centers for Disease Control and Prevention; 2000. http://www.cdc.gov/ HRQOL/pdfs/mhd.pdf. Accessed August 24, 2015.

[14] Brown DW , Balluz LS , Heath GW , Moriarty DG , Ford ES , Giles WH , Associations between recommended levels of physical activity and health-related quality of life. Findings from the 2001 Behavioral Risk Factor Surveillance System (BRFSS) survey. Prev Med 2003;37(5):520–8. 10.1016/S0091-7435(03)00179-8 14572437

[15] Moore LV , Dodd KW , Thompson FE , Grimm KA , Kim SA , Scanlon KS . Using Behavioral Risk Factor Surveillance System data to estimate the percent of the population meeting USDA food patterns fruit and vegetable intake recommendations. Am J Epidemiol 2015;181(12):979–88. 10.1093/aje/kwu461 25935424 PMC4465876

[16] Bieler GS , Brown GG , Williams RL , Brogan DJ . Estimating model-adjusted risks, risk differences, and risk ratios from complex survey data. Am J Epidemiol 2010;171(5):618–23. 10.1093/aje/kwp440 20133516

[17] Reuben DB , Tinetti ME . Goal-oriented patient care—an alternative health outcomes paradigm. N Engl J Med 2012;366(9):777–9. 10.1056/NEJMp1113631 22375966

[18] Mody RR , Smith MJ . Smoking status and health-related quality of life: as findings from the 2001 Behavioral Risk Factor Surveillance System data. Am J Health Promot 2006;20(4):251–8. 10.4278/0890-1171-20.4.251 16555798

[19] Allen NB , Badon S , Greenlund KJ , Huffman M , Hong Y , Lloyd-Jones DM . The association between cardiovascular health and health-related quality of life and health status measures among U.S. adults: a cross-sectional study of the National Health and Nutrition Examination Surveys, 2001-2010. Health Qual Life Outcomes 2015;13(1):152. 10.1186/s12955-015-0352-z 26396070 PMC4580297

[20] Dutton GR , Lewis CE . The Look AHEAD trial: implications for lifestyle intervention in type 2 diabetes mellitus. Prog Cardiovasc Dis 2015;58(1):69–75. 10.1016/j.pcad.2015.04.002 25936906 PMC4501472

[21] Espeland MA , Glick HA , Bertoni A , Brancati FL , Bray GA , Clark JM , ; Look AHEAD Research Group. Impact of an intensive lifestyle intervention on use and cost of medical services among overweight and obese adults with type 2 diabetes: the action for health in diabetes. Diabetes Care 2014;37(9):2548–56. 10.2337/dc14-0093 25147253 PMC4140155

[22] Bowlin SJ , Morrill BD , Nafziger AN , Jenkins PL , Lewis C , Pearson TA . Validity of cardiovascular disease risk factors assessed by telephone survey: the Behavioral Risk Factor Survey. J Clin Epidemiol 1993;46(6):561–71. 10.1016/0895-4356(93)90129-O 8501483

[23] Fahimi M , Link M , Mokdad A , Schwartz DA , Levy P . Tracking chronic disease and risk behavior prevalence as survey participation declines: statistics from the behavioral risk factor surveillance system and other national surveys. Prev Chronic Dis 2008;5(3):A80. 18558030 PMC2483564

[24] Dauchet L , Amouyel P , Hercberg S , Dallongeville J . Fruit and vegetable consumption and risk of coronary heart disease: a meta-analysis of cohort studies. J Nutr 2006;136(10):2588–93. 16988131 10.1093/jn/136.10.2588

